# Dioxidobis(2-oxo-1,2-dihydropyridin-3-olato)­molybdenum(VI)

**DOI:** 10.1107/S1600536808007782

**Published:** 2008-03-29

**Authors:** Manoj Trivedi, Daya Shankar Pandey, Nigam P. Rath

**Affiliations:** a Department of Chemistry, Faculty of Science, Banaras Hindu University, Varanasi 221005, India; b Department of Chemistry and Biochemistry and Center for Nanoscience, University of Missouri-St Louis, One University Boulevard, St Louis, MO 63121-4499, USA

## Abstract

In the title compound, [Mo(C_5_H_4_NO_2_)_2_O_2_], the Mo^VI^ atom exhibits a distorted octa­hedral coordination geometry formed by two terminal oxo ligands and two monoanionic *O*,*O*-bidentate pyridinone ligands. The two terminal oxo ligands lie in a *cis* arrangement, the ketonic O atoms of the pyridinone ligands are coordinated *trans* to the oxo ligands and the deprotonated hydroxyl O atoms are located *trans* to each other. The crystal structure contains inter­molecular N—H⋯O hydrogen bonds, C—H⋯O contacts and face-to-face π–π stacking inter­actions with an inter­planar separation of 3.25 (1) Å.

## Related literature

For general background, see: Veiros *et al.* (2006 ▶); Tucci *et al.* (1998 ▶); Collison *et al.* (1996 ▶); Hille (1996 ▶). For related structures, see: Brown *et al.* (2004 ▶); Hanna *et al.* (2000 ▶); Thompson *et al.* (1999 ▶); Zhang *et al.* (1992 ▶). For related literature, see: Braga *et al.* (1997 ▶); Grasselli (1999 ▶); Hozba *et al.* (1997 ▶); Ranganathan *et al.* (1998 ▶); Schrock (1998 ▶); Schultz *et al.* (1993 ▶). 
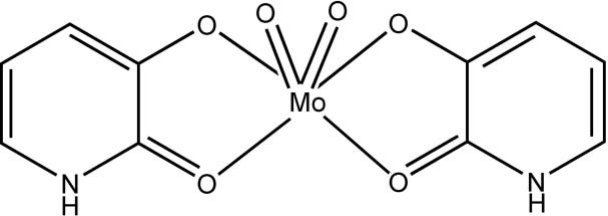


## Experimental

### 

#### Crystal data

[Mo(C_5_H_4_NO_2_)_2_O_2_]
*M* *_r_* = 348.12
Monoclinic, 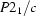

*a* = 13.263 (3) Å
*b* = 7.2470 (14) Å
*c* = 13.264 (3) Å
β = 118.540 (9)°
*V* = 1120.0 (4) Å^3^
*Z* = 4
Mo *K*α radiation
μ = 1.20 mm^−1^
*T* = 100 (2) K
0.29 × 0.16 × 0.09 mm

#### Data collection

Bruker APEXII CCD diffractometer
Absorption correction: multi-scan (*SADABS*; Sheldrick, 2007 ▶) *T*_min_ = 0.723, *T*_max_ = 0.899
37847 measured reflections
3123 independent reflections
2772 reflections with *I* > 2σ(*I*)
*R*_int_ = 0.044

#### Refinement

*R*[*F* ^2^ > 2σ(*F* ^2^)] = 0.025
*wR*(*F* ^2^) = 0.058
*S* = 1.08
3123 reflections
172 parameters
H-atom parameters constrained
Δρ_max_ = 0.70 e Å^−3^
Δρ_min_ = −0.52 e Å^−3^

### 

Data collection: *APEX2* (Bruker, 2006 ▶); cell refinement: *SAINT* (Bruker, 2006 ▶); data reduction: *SAINT*; program(s) used to solve structure: *SHELXS97* (Sheldrick, 2008 ▶); program(s) used to refine structure: *SHELXL97* (Sheldrick, 2008 ▶); molecular graphics: *SHELXL97*; software used to prepare material for publication: *SHELXL97*.

## Supplementary Material

Crystal structure: contains datablocks global, I. DOI: 10.1107/S1600536808007782/bi2282sup1.cif

Structure factors: contains datablocks I. DOI: 10.1107/S1600536808007782/bi2282Isup2.hkl

Additional supplementary materials:  crystallographic information; 3D view; checkCIF report

## Figures and Tables

**Table:** 

Mo1—O1	1.9972 (14)
Mo1—O2	2.1886 (15)
Mo1—O3	1.9790 (14)
Mo1—O4	2.1882 (15)
Mo1—O5	1.7062 (15)
Mo1—O6	1.7124 (16)

**Table:** 

O5—Mo1—O6	103.48 (7)

**Table 2:** Hydrogen-bond geometry (Å, °)

*D*—H⋯*A*	*D*—H	H⋯*A*	*D*⋯*A*	*D*—H⋯*A*
N1—H1N⋯O5^i^	0.88	2.16	2.900 (2)	142
N2—H2N⋯O6^ii^	0.88	1.91	2.776 (3)	167
C3—H3⋯O6^iii^	0.95	2.51	3.428 (3)	162
C9—H9⋯O2^iv^	0.95	2.38	3.235 (3)	150

Symmetry codes: (i) 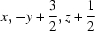; (ii) 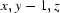; (iii) 
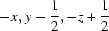
; (iv) 
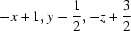
.

**Fig. 1.**
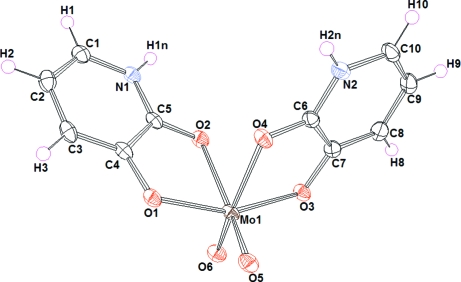
The molecular structure with displacement ellipsoids drawn at the 50% probability level for non-H atoms

**Fig. 2.**
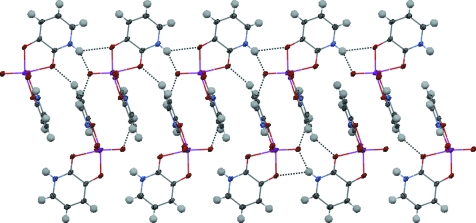
Parallel chains made through intermolecular N—H···O and C—H···O hydrogen-bond interactions (dashed lines)

**Fig. 3.**
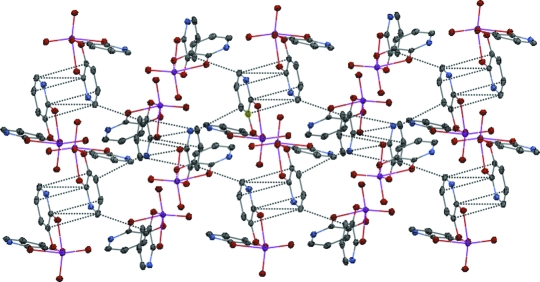
Face-to-face π-π interactions

**Fig. 4.**
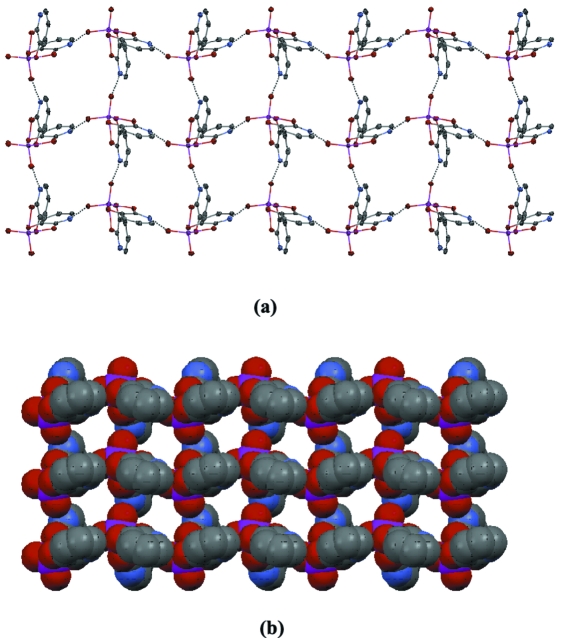
Views along the a axis

## supplementary crystallographic information

### Comment

There has been growing interest in the study of Mo^VI^ complexes because of their biochemical significance (Collison *et al.*, 1996; Hille, 1996). For example, dioxomolybdenum(VI) complexes are studied as models for oxidized forms of molybdoenzymes, *e.g. *aldehyde oxidase and sulfite oxidase which are supposed to contain *cis*-Mo*X*_2_ units (*X* = O,*S*) coordinated to S, N and O donor atoms of the protein structure (Tucci *et al.*, 1998; Schultz *et al.*, 1993). The present view of these enzymes indicates that the formal oxidation state of Mo cycles between +4 and +6 in reactions with substrate and oxidant. The two electron O atom transfer seems to be the relevant mechanism in understanding the chemical role of enzymatic reactions. A large number of important chemical reactions are catalysed by Mo^VI^ complexes. Several industrial processes such as ammoxidation of propene to acrylonitrile (Grasselli, 1999), olefin epoxidation (Veiros *et al.*, 2006) and olefin metathesis (Schrock, 1998) reactions are carried out over Mo catalysts.

In the title compound, the coordination sphere about the Mo^VI^ atom consists of six O atoms arranged in a distorted octahedral geometry (Fig. 1 and Table 1). There is a *cis* arrangement of dioxo ligands, as predicted by spectroscopic and other structural data. The O=Mo=O angle is 103.48 (7) and the Mo=O distances are 1.706 (15) and 1.712 (16) Å [average 1.709 (15) Å], comparable to those found in other *cis*-dioxomolybdenum(VI) complexes (Hanna *et al.*, 2000; Brown *et al.*, 2004). The two ketonic O atoms of the pyridinone ligands are *trans* to the oxo ligands and the stronger field hydroxyl O atoms are *trans* to one another. As expected, a slight lengthening of the ketone C=O bond is observed upon complexation, with a mean distance of 1.272 (2) Å, and the Mo—O(ketone) bonds [average 2.188 (15) Å] are somewhat longer than the Mo—*O*(hydroxyl) distances [average 1.988 (14) Å]. A pronounced localization of the formal double bonds in the pyridinone rings is clearly indicated by the short C1—C2 and C9—C10 bonds [average 1.360 (3) Å], long C4—C5 and C6—C7 bonds [average 1.425 (3) Å], and short ketone C5—O2 and C6—O4 [average 1.272 (2) Å] bonds. Resonance forms for pyridinone ligands have been described in detail elsewhere (Thompson *et al.*, 1999; Zhang *et al.*, 1992).

The NH and CH groups of the pyridinone ligands form a hydrogen bond with an oxo ligand attached to Mo in a neighbouring molecule (Table 2) (Braga *et al.*, 1997). Repetition of this hydrogen bond generates parallel chains along the *b* axis (Fig. 2). There are face-to-face π–π stacking interactions involving the pyridinone rings of adjacent pyridinone molecules, with π–π distances of 3.295–3.389 Å (Fig. 3) (Ranganathan *et al.*, 1998; Hozba *et al.*, 1997). One potential driving force for alignment of the motifs might be the N···O interactions (N···O distance = 2.904 Å) that exists between adjacent motifs, resulting in a columnar architecture with a dimension of 7.2 × 6.7 Å (Fig. 4).

### Experimental

The title compound was prepared by suspension of 2,3-pyridinediol (0.111 g, 1 mmol) in methanol (30 ml), followed by addition of KOH (0.112 g, 2 mmol). Stirring at room temperature for 30 min gave a clear red solution. This solution was treated with (NH_4_)_2_Mo_2_O_7_ (0.170 g, 0.50 mmol) and stirred overnight. The resulting orange-red solution was filtered and allowed to cool at room temperature. Over a couple of days, orange irregular needle-shaped diffraction-quality crystals separated, which were isolated and dried in air.

### Refinement

All H atoms were added in calculated positions and were refined as riding with *U*_iso_(H) = 1.2*U*_eq_(C).

### Crystal data

**Table:**

[Mo(C_5_H_4_NO_2_)_2_O_2_]	*F*_000_ = 688
*M**_r_* = 348.12	*D*_x_ = 2.065 Mg m^−^^3^*D*_m_ = no Mg m^−^^3^*D*_m_ measured by not measured
Monoclinic, *P*2_1_/*c*	Mo *K*α radiation λ = 0.71073 Å
Hall symbol: -P 2ybc	Cell parameters from 9899 reflections
*a* = 13.263 (3) Å	θ = 1.8–29.6º
*b* = 7.2470 (14) Å	µ = 1.20 mm^−^^1^
*c* = 13.264 (3) Å	*T* = 100 (2) K
β = 118.540 (9)º	Needle, orange
*V* = 1120.0 (4) Å^3^	0.29 × 0.16 × 0.09 mm
*Z* = 4	

### Data collection

**Table:**

Bruker APEXII CCD diffractometer	3123 independent reflections
Radiation source: fine-focus sealed tube	2772 reflections with *I* > 2σ(*I*)
Monochromator: graphite	*R*_int_ = 0.045
*T* = 100(2) K	θ_max_ = 29.6º
φ and ω scans	θ_min_ = 1.8º
Absorption correction: multi-scan(SADABS; Sheldrick, 2007)	*h* = −18→18
*T*_min_ = 0.723, *T*_max_ = 0.899	*k* = −10→10
37847 measured reflections	*l* = −18→18

### Refinement

**Table:**

Refinement on *F*^2^	Secondary atom site location: difference Fourier map
Least-squares matrix: full	Hydrogen site location: inferred from neighbouring sites
*R*[*F*^2^ > 2σ(*F*^2^)] = 0.025	H-atom parameters constrained
*wR*(*F*^2^) = 0.058	*w* = 1/[σ^2^(*F*_o_^2^) + (0.0224*P*)^2^ + 1.1984*P*] where *P* = (*F*_o_^2^ + 2*F*_c_^2^)/3
*S* = 1.08	(Δ/σ)_max_ = 0.001
3123 reflections	Δρ_max_ = 0.70 e Å^−^^3^
172 parameters	Δρ_min_ = −0.52 e Å^−^^3^
Primary atom site location: structure-invariant direct methods	Extinction correction: none

### Special details

**Table:**

Geometry. All e.s.d.'s (except the e.s.d. in the dihedral angle between two l.s. planes) are estimated using the full covariance matrix. The cell e.s.d.'s are taken into account individually in the estimation of e.s.d.'s in distances, angles and torsion angles; correlations between e.s.d.'s in cell parameters are only used when they are defined by crystal symmetry. An approximate (isotropic) treatment of cell e.s.d.'s is used for estimating e.s.d.'s involving l.s. planes.
Refinement. Refinement of *F*^2^ against ALL reflections. The weighted *R*-factor *wR* and goodness of fit *S* are based on *F*^2^, conventional *R*-factors *R* are based on *F*, with *F* set to zero for negative *F*^2^. The threshold expression of *F*^2^ > σ(*F*^2^) is used only for calculating *R*-factors(gt) *etc*. and is not relevant to the choice of reflections for refinement. *R*-factors based on *F*^2^ are statistically about twice as large as those based on *F*, and *R*- factors based on ALL data will be even larger.

### Fractional atomic coordinates and isotropic or equivalent isotropic displacement parameters (Å^2^)

**Table:**

	*x*	*y*	*z*	*U*_iso_*/*U*_eq_	
Mo1	0.248605 (14)	0.91497 (2)	0.414412 (14)	0.01513 (6)	
O1	0.08144 (11)	0.8541 (2)	0.33241 (11)	0.0172 (3)	
O2	0.20602 (12)	0.8648 (2)	0.55242 (12)	0.0176 (3)	
O3	0.40951 (12)	0.8995 (2)	0.53830 (12)	0.0173 (3)	
O4	0.27660 (12)	0.6185 (2)	0.44705 (12)	0.0177 (3)	
O5	0.25461 (12)	0.8903 (2)	0.28946 (12)	0.0196 (3)	
O6	0.25285 (12)	1.1491 (2)	0.43339 (12)	0.0204 (3)	
N1	0.06163 (14)	0.7521 (2)	0.58314 (14)	0.0166 (3)	
H1N	0.1075	0.7516	0.6578	0.020*	
N2	0.41302 (15)	0.4130 (2)	0.56670 (15)	0.0181 (3)	
H2N	0.3655	0.3201	0.5347	0.022*	
C1	−0.04904 (17)	0.6950 (3)	0.54263 (18)	0.0185 (4)	
H1	−0.0754	0.6548	0.5942	0.022*	
C2	−0.12129 (17)	0.6964 (3)	0.42750 (18)	0.0206 (4)	
H2	−0.1989	0.6594	0.3987	0.025*	
C3	−0.08198 (17)	0.7519 (3)	0.35098 (17)	0.0189 (4)	
H3	−0.1325	0.7526	0.2706	0.023*	
C4	0.02965 (17)	0.8049 (3)	0.39344 (16)	0.0160 (4)	
C5	0.10352 (16)	0.8086 (3)	0.51457 (16)	0.0148 (4)	
C6	0.37661 (17)	0.5850 (3)	0.52910 (16)	0.0157 (4)	
C7	0.45352 (17)	0.7346 (3)	0.58207 (16)	0.0166 (4)	
C8	0.56213 (17)	0.7023 (3)	0.66842 (17)	0.0203 (4)	
H8	0.6140	0.8016	0.7037	0.024*	
C9	0.59590 (18)	0.5190 (3)	0.70425 (18)	0.0231 (5)	
H9	0.6710	0.4941	0.7645	0.028*	
C10	0.52126 (18)	0.3776 (3)	0.65289 (18)	0.0218 (4)	
H10	0.5444	0.2542	0.6770	0.026*	

### Atomic displacement parameters (Å^2^)

**Table:**

	*U*^11^	*U*^22^	*U*^33^	*U*^12^	*U*^13^	*U*^23^
Mo1	0.01552 (9)	0.01402 (9)	0.01085 (9)	0.00111 (6)	0.00226 (6)	0.00057 (6)
O1	0.0157 (6)	0.0197 (7)	0.0102 (6)	0.0007 (6)	0.0014 (5)	0.0007 (5)
O2	0.0159 (6)	0.0189 (7)	0.0120 (6)	−0.0009 (6)	0.0019 (5)	−0.0005 (5)
O3	0.0148 (6)	0.0167 (7)	0.0146 (7)	−0.0010 (5)	0.0023 (5)	0.0004 (5)
O4	0.0166 (6)	0.0152 (7)	0.0150 (7)	0.0016 (5)	0.0025 (5)	0.0001 (5)
O5	0.0200 (7)	0.0226 (8)	0.0122 (7)	0.0020 (6)	0.0045 (6)	0.0012 (6)
O6	0.0233 (7)	0.0158 (7)	0.0198 (7)	0.0012 (6)	0.0084 (6)	0.0010 (6)
N1	0.0181 (8)	0.0156 (8)	0.0113 (7)	0.0011 (7)	0.0031 (6)	−0.0005 (7)
N2	0.0213 (8)	0.0172 (8)	0.0168 (8)	0.0037 (7)	0.0099 (7)	0.0026 (7)
C1	0.0205 (10)	0.0137 (9)	0.0201 (10)	0.0017 (8)	0.0086 (8)	−0.0009 (8)
C2	0.0162 (9)	0.0193 (10)	0.0217 (10)	0.0000 (8)	0.0053 (8)	−0.0043 (8)
C3	0.0155 (9)	0.0184 (10)	0.0143 (9)	0.0021 (8)	0.0003 (7)	−0.0031 (8)
C4	0.0183 (9)	0.0122 (9)	0.0120 (9)	0.0028 (7)	0.0028 (7)	−0.0011 (7)
C5	0.0166 (9)	0.0089 (8)	0.0131 (9)	0.0017 (7)	0.0025 (7)	−0.0016 (7)
C6	0.0168 (9)	0.0183 (10)	0.0117 (9)	0.0024 (8)	0.0066 (7)	0.0016 (7)
C7	0.0172 (9)	0.0209 (10)	0.0112 (9)	0.0016 (8)	0.0065 (7)	0.0003 (8)
C8	0.0168 (9)	0.0312 (12)	0.0114 (9)	0.0001 (8)	0.0055 (8)	0.0001 (8)
C9	0.0178 (9)	0.0384 (13)	0.0115 (9)	0.0088 (9)	0.0057 (8)	0.0072 (9)
C10	0.0236 (10)	0.0270 (11)	0.0158 (10)	0.0114 (9)	0.0103 (8)	0.0091 (8)

### Geometric parameters (Å, °)

**Table:**

Mo1—O1	1.9972 (14)	N2—H2N	0.880
Mo1—O2	2.1886 (15)	C1—C2	1.361 (3)
Mo1—O3	1.9790 (14)	C1—H1	0.950
Mo1—O4	2.1882 (15)	C2—C3	1.403 (3)
Mo1—O5	1.7062 (15)	C2—H2	0.950
Mo1—O6	1.7124 (16)	C3—C4	1.364 (3)
O1—C4	1.336 (3)	C3—H3	0.950
O2—C5	1.270 (2)	C4—C5	1.427 (3)
O3—C7	1.335 (2)	C6—C7	1.423 (3)
O4—C6	1.274 (2)	C7—C8	1.366 (3)
N1—C5	1.337 (3)	C8—C9	1.410 (3)
N1—C1	1.364 (3)	C8—H8	0.950
N1—H1N	0.880	C9—C10	1.360 (3)
N2—C6	1.344 (3)	C9—H9	0.950
N2—C10	1.366 (3)	C10—H10	0.950
			
O5—Mo1—O6	103.48 (7)	C1—C2—C3	120.52 (19)
O5—Mo1—O3	105.57 (7)	C1—C2—H2	119.7
O6—Mo1—O3	89.27 (6)	C3—C2—H2	119.7
O5—Mo1—O1	90.16 (6)	C4—C3—C2	119.16 (18)
O6—Mo1—O1	104.40 (6)	C4—C3—H3	120.4
O3—Mo1—O1	156.33 (6)	C2—C3—H3	120.4
O5—Mo1—O4	90.40 (6)	O1—C4—C3	126.57 (18)
O6—Mo1—O4	162.43 (6)	O1—C4—C5	113.69 (17)
O3—Mo1—O4	76.49 (6)	C3—C4—C5	119.74 (19)
O1—Mo1—O4	86.00 (6)	O2—C5—N1	122.93 (17)
O5—Mo1—O2	161.00 (6)	O2—C5—C4	118.70 (18)
O6—Mo1—O2	92.59 (6)	N1—C5—C4	118.37 (18)
O3—Mo1—O2	84.43 (6)	O4—C6—N2	122.44 (19)
O1—Mo1—O2	75.87 (6)	O4—C6—C7	119.02 (18)
O4—Mo1—O2	76.04 (5)	N2—C6—C7	118.53 (18)
C4—O1—Mo1	119.16 (12)	O3—C7—C8	125.8 (2)
C5—O2—Mo1	112.20 (12)	O3—C7—C6	113.95 (17)
C7—O3—Mo1	119.05 (12)	C8—C7—C6	120.3 (2)
C6—O4—Mo1	111.40 (13)	C7—C8—C9	118.8 (2)
C5—N1—C1	122.94 (17)	C7—C8—H8	120.6
C5—N1—H1N	118.5	C9—C8—H8	120.6
C1—N1—H1N	118.5	C10—C9—C8	120.20 (19)
C6—N2—C10	122.19 (19)	C10—C9—H9	119.9
C6—N2—H2N	118.9	C8—C9—H9	119.9
C10—N2—H2N	118.9	C9—C10—N2	120.0 (2)
C2—C1—N1	119.2 (2)	C9—C10—H10	120.0
C2—C1—H1	120.4	N2—C10—H10	120.0
N1—C1—H1	120.4		
			
O5—Mo1—O1—C4	162.99 (15)	C2—C3—C4—O1	177.13 (19)
O6—Mo1—O1—C4	−93.04 (15)	C2—C3—C4—C5	−1.9 (3)
O3—Mo1—O1—C4	30.6 (2)	Mo1—O2—C5—N1	173.36 (15)
O4—Mo1—O1—C4	72.60 (14)	Mo1—O2—C5—C4	−6.4 (2)
O2—Mo1—O1—C4	−3.99 (13)	C1—N1—C5—O2	178.72 (18)
O5—Mo1—O2—C5	−38.2 (3)	C1—N1—C5—C4	−1.5 (3)
O6—Mo1—O2—C5	109.76 (14)	O1—C4—C5—O2	3.4 (3)
O3—Mo1—O2—C5	−161.22 (14)	C3—C4—C5—O2	−177.45 (18)
O1—Mo1—O2—C5	5.55 (13)	O1—C4—C5—N1	−176.45 (17)
O4—Mo1—O2—C5	−83.79 (13)	C3—C4—C5—N1	2.7 (3)
O5—Mo1—O3—C7	−88.95 (15)	Mo1—O4—C6—N2	178.53 (15)
O6—Mo1—O3—C7	167.25 (15)	Mo1—O4—C6—C7	−2.4 (2)
O1—Mo1—O3—C7	41.0 (2)	C10—N2—C6—O4	178.42 (19)
O4—Mo1—O3—C7	−2.36 (14)	C10—N2—C6—C7	−0.6 (3)
O2—Mo1—O3—C7	74.58 (14)	Mo1—O3—C7—C8	−179.11 (16)
O5—Mo1—O4—C6	108.44 (14)	Mo1—O3—C7—C6	1.9 (2)
O6—Mo1—O4—C6	−34.2 (3)	O4—C6—C7—O3	0.6 (3)
O3—Mo1—O4—C6	2.52 (13)	N2—C6—C7—O3	179.69 (17)
O1—Mo1—O4—C6	−161.43 (14)	O4—C6—C7—C8	−178.48 (19)
O2—Mo1—O4—C6	−85.01 (14)	N2—C6—C7—C8	0.6 (3)
C5—N1—C1—C2	−0.6 (3)	O3—C7—C8—C9	−179.41 (19)
N1—C1—C2—C3	1.4 (3)	C6—C7—C8—C9	−0.4 (3)
C1—C2—C3—C4	−0.1 (3)	C7—C8—C9—C10	0.3 (3)
Mo1—O1—C4—C3	−177.08 (16)	C8—C9—C10—N2	−0.3 (3)
Mo1—O1—C4—C5	2.0 (2)	C6—N2—C10—C9	0.5 (3)

### Hydrogen-bond geometry (Å, °)

**Table:**

*D*—H···*A*	*D*—H	H···*A*	*D*···*A*	*D*—H···*A*
N1—H1N···O5^i^	0.88	2.16	2.900 (2)	142
N2—H2N···O6^ii^	0.88	1.91	2.776 (3)	167
C3—H3···O6^iii^	0.95	2.51	3.428 (3)	162
C9—H9···O2^iv^	0.95	2.38	3.235 (3)	150

Symmetry codes: (i) *x*, −*y*+3/2, *z*+1/2; (ii) *x*, *y*−1, *z*; (iii) −*x*, *y*−1/2, −*z*+1/2; (iv) −*x*+1, *y*−1/2, −*z*+3/2.
